# Quantitative Characterization by Transmission Electron Microscopy and Its Application to Interfacial Phenomena in Crystalline Materials

**DOI:** 10.3390/ma17030578

**Published:** 2024-01-25

**Authors:** Seiichiro Ii

**Affiliations:** Research Center for Structural Materials, National Institute for Materials Science (NIMS), Tsukuba 305-0047, Japan; ii.seiichiro@nims.go.jp

**Keywords:** transmission electron microscopy, energy dispersive X-ray spectroscopy, electron energy loss spectroscopy, grain boundary, interphase boundary

## Abstract

This paper reviews quantitative characterization via transmission electron microscopy (TEM) and its application to interfacial phenomena based on the results obtained through the studies. Several signals generated by the interaction between the specimen and the electron beam with a probe size of less than 1 nm are utilized for a quantitative analysis, which yields considerable chemical and physical information. This review describes several phenomena near the interfaces, e.g., clear solid–vapor interface (surface) segregation of yttria in the zirconia nanoparticles by an energy-dispersive X-ray spectroscopy analysis, the evaluation of the local magnetic moment at the grain boundary in terms of electron energy loss spectroscopy equipped with TEM, and grain boundary character dependence of the magnetism. The direct measurement of the stress to the dislocation transferred across the grain boundary and the microstructure evolution focused on the grain boundary formation caused by plastic deformation are discussed as examples of material dynamics associated with the grain boundary. Finally, the outlook for future investigations of interface studies, including the recent progress, is also discussed.

## 1. Introduction

The microstructures of materials need to be designed and controlled at the nanometer scale to satisfy the recent increase in the demand for engineering materials. Further, the material response to an external field and the corresponding microstructure need to be understood quantitatively for the theoretical modeling of material behavior.

**Figure 1:**
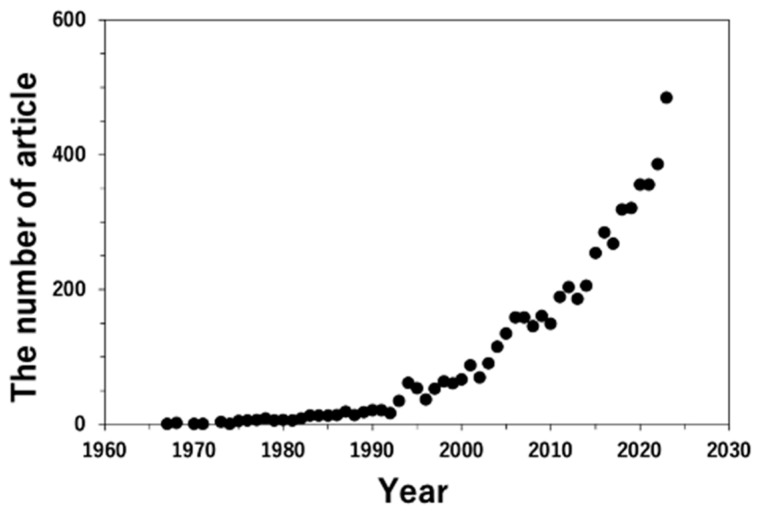
The change in the number of articles in the fields of “Materials Science”, “Physics and Astronomy”, “Chemistry”, and “Engineering”, which include “quantitative analysis”, and “transmission electron microscopy” or “TEM”, in the title or abstract, or keywords. Data are referred to by scopus.com (accessed on 12 September 2023).

Transmission electron microscopy (TEM) [1] and scanning electron microscopy (SEM) [2] have developed and become commercially available, and microstructure characterization has remarkably progressed in science and technology at the sub-micrometer and nanometer scales. The advantage of electron microscopy over optical microscopy is the use of electrons, which result in an interaction with a specimen. An electron beam provides a high resolution because of its considerably shorter wavelength than light, and electron diffraction can be obtained from crystallographic and geometric information. Further, the emitted X-rays and inelastic electrons, in which the initial energy is lost because of the interaction, are characterized by spectroscopy, i.e., energy-dispersive X-ray spectroscopy (EDS) and electron energy-loss spectroscopy (EELS). Chemical and physical properties, such as the identification of elements and their composition are analyzed by EDS and the valency of ions and an electronic structure are analyzed by EELS. Detailed information on TEM can be found in well-known textbooks [3,4,5,6,7,8]. Moreover, the acquisition of digital data using a charge-coupled device (CCD) [9,10,11] and an imaging plate (IP) film [12,13,14,15,16,17] enables a more efficient quantitative analysis with analytical tools such as EDS and EELS. Figure 1 illustrates the number of articles, including the words “quantitative analysis” and “transmission electron microscopy (or TEM)” in the “title”, “abstract”, or “keywords”. This was referred to by the Scopus [18]. The number of papers has increased steeply since the early 1990s, which is the incipient time in which reports on the CCD and IP were published in the same era. Further, this progress is one of the reasons why quantitative analysis using TEM has been widely conducted.

In this article, the quantification analyses by TEM are presented based on some examples from the author’s experiments to highlight their application in the interfaces. The interface, known as an interphase or grain boundary, is a two-dimensional lattice defect in polycrystalline and multiphase materials [19,20,21,22]. The interfaces are high-energy states in comparison with the matrix as a defect-free region, which results in different chemical and physical behaviors. Further, they often govern their mechanical and functional properties and play an important role from an engineering viewpoint [19,21,22,23,24]. Many researchers have attempted to control the grain boundary microstructure to improve its properties based on the concept of “grain boundary engineering” proposed by Watanabe [25,26]. Understanding the structure (both atomic and chemical) and properties of individual interfaces and the relationship between them is essential for controlling the grain boundary and interface structure. Therefore, the nature of the interfaces needs to be understood from a scientific viewpoint for engineering applications. As for studies on grain boundaries and interfaces, the international conference on the intergranular and interphase boundary in materials (IIB) is held every three years [27]. The papers from the recent IIB after 2004 are published as the special issue [28,29,30,31,32,33,34], and it can be pursued as the trend of the research on grain boundaries and interfaces.

This paper reviews the microstructural characterizations of several interfaces using TEM equipped with EDS and EELS. In the next section, the chemical characterization of TEM-EDS is described with application to the solid–vapor (surface) segregation of the zirconia nanoparticle, in which the sample preparation is contrived to analyze the whole of the individual particles. Then, the local magnetic moment characterization at the grain boundary is explained as physical properties characterized by EELS as one of the new characterization techniques. Recently, scanning TEM (STEM) has been recognized as a powerful tool, and both EDS and EELS analyses combined with STEM have become the mainstream for quantitative characterization on a nanoscale. Particularly, a STEM-equipped aberration correction system enables characterization on an atomic scale. However, this article focuses on the TEM characterization that has been conducted so far, and the quantitative characterization of STEM is described later in the section of prospects. The dynamics of the interfaces investigated by in situ straining TEM experiments are also presented. In performing the in situ experiments, the direct measurement succeeded in the dislocation transfer to the grain boundary. Moreover, the dynamics of the microstructure evolved to form grain boundaries during plastic deformation. SEM, which is frequently used for characterizing the microstructures [35,36], and the capability of combining the grain boundary character distribution with electron backscattered diffraction (EBSD), is discussed in detail [37,38,39]. However, the review only discusses the characterization of the interface and the grain boundary by TEM as the other points are beyond the scope of the review.

## 2. Solid–Vapor Interface (Surface) Segregation Analyzed by TEM-EDS

EDS is commonly used to identify the elements existing in a particular area, and it is one of the most popular attachments equipped with TEM and SEM. Thus far, several textbooks on EDS have been published [5,8,35]. The principles of the quantitative analysis are briefly explained in this section. The inner-shell electron is ejected at a specific energy when the incident electron probe penetrates the specimen. The ionized atom returns to a stable (ground) state by filling the electron from the outer shell because the ionized atom, in which the electron in the inner shell is unoccupied, is known as the excited state. The signal emitted upon returning to the ground state is an X-ray, and the energy-ejecting electrons depend on the elements. Therefore, it is referred to as a characteristic X-ray. The signal was transformed into a current from the detected photons by a detector (converter), and the EDS system displayed the signal as a profile. A quantitative profile, such as the composition of elements *x*, *C_x_* was evaluated by calculating the peak ratio expressed as

(1) $$C_{A}/C_{B}=K_{AB}\times I_{A}/I_{B},$$

where *I_x_* represents the peak intensity of element *x*. Equation (1) is the basis for the quantitative EDS analysis. Therefore, it was used in both SEM (bulk specimen) and TEM (thin-film specimen). The coefficient *K_AB_* includes the entire process from the X-ray emission to the display as the signal. Consequently, calibration processes are conducted for quantitative evaluations [5,35,40,41]. The thin-film approximation, which excludes the X-ray absorption in the specimen, is generally used for TEM-EDS [5]. As an improved quantitative chemical analysis, the ζ factor analysis proposed by Watanabe has recently gained significant research attention for the quantitative EDS analysis to improve the accuracy of quantities [40,41]. Further, the X-ray efficiency is improved significantly by the configuration of the specimen and X-ray detector and by the enlargement of the detector [42]. Further, the EDS analysis is frequently conducted using aberration (Cs)-corrected scanning transmission electron microscopy (STEM) and chemical analysis on an atomic scale with the simultaneous observation of the atomic structure [43,44,45,46,47,48,49,50]. Several studies on this topic are currently underway [44,45,46,47], but this technique expands our knowledge of the chemical distribution of elements, such as grain boundary segregation in ceramic materials with highly ordered structures [48,49] and metallic materials [50]. In this section, as a case study of the application of EDS, the quantitative analysis around a solid–vapor interface in zirconia (ZrO_2_) nanoparticles, which includes yttria (Y_2_O_3_), i.e., the distribution of Y_2_O_3_ near the surface of the nanoparticle, is examined [51].

**Figure 2:**
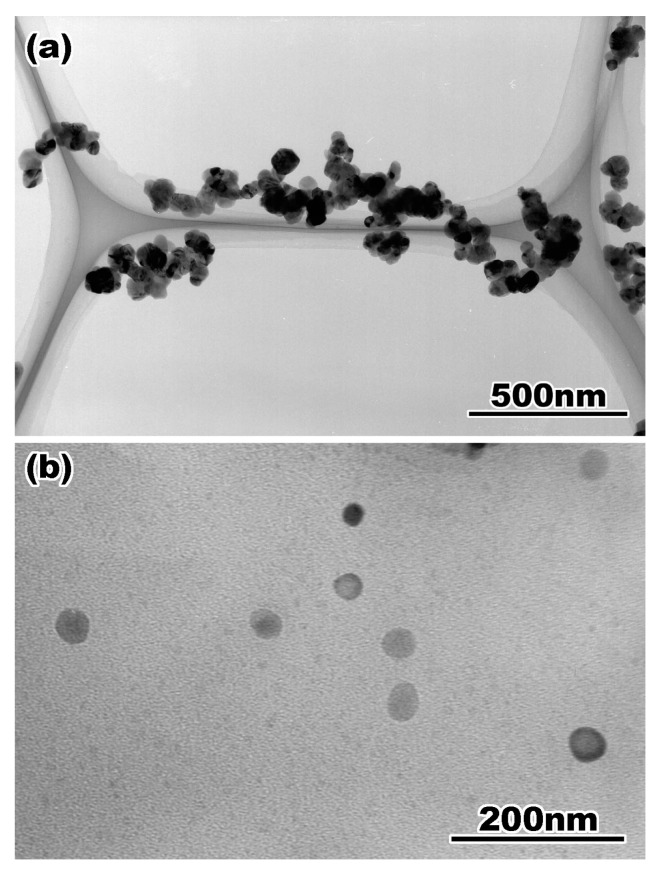
A bright field image of yttria doped zirconia particles taken from the TEM specimens prepared by (**a**) common method and (**b**) cross-sectional method. Reprinted from Ref. [51] with permission from John Wiley and Sons.

Figure 2a,b show TEM bright-field images of the ZrO_2_ nanoparticles prepared by the (a) common method and (b) ion milling method for cross-sectional observation. In the conventional method, a mixture of ZrO_2_ powder and ethanol is added to a TEM grid. The proposed method is simple and easy to implement; however, because of particle aggregation, the particles overlap with each other, as shown in Figure 2a, and it is difficult to observe the isolated particles. For the cross-sectional observation, particles dispersed ultrasonically are mixed with the epoxy resin and prepared by the standard method of mechanical grinding, dimpling, and ion milling. For the particle–resin mixture, the ZrO_2_ particles in the TEM specimen are smaller; however, they can be observed individually, as shown in Figure 2b.

**Figure 3:**
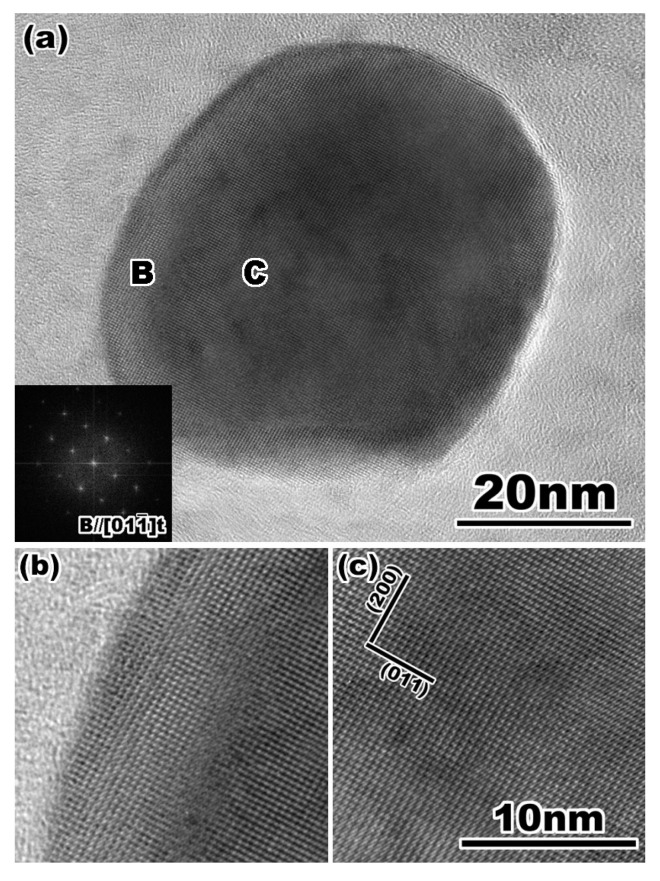
(**a**) A high-resolution transmission electron micrograph of yttria doped zirconia particle. The FFT pattern in (**a**) indicates that the particle is tetragonal single phase. Enlarged lattice image of (**b**) the surface and (**c**) the internal region of the particle indicated by B and C in (**a**), respectively. Reprinted from Ref. [51] with permission from John Wiley and Sons.

Figure 3 shows a high-resolution TEM image of ZrO_2_ particles. The entire particle is flattened by the ion milling process, and the atomic structure inside the particle in Figure 3c, as well as the surface region shown in Figure 3b, is observed clearly. Furthermore, the ion-milled particles are chemically analyzed without considering shadowing because of the rounded shape and uneven surface of the specimen.

**Figure 4:**
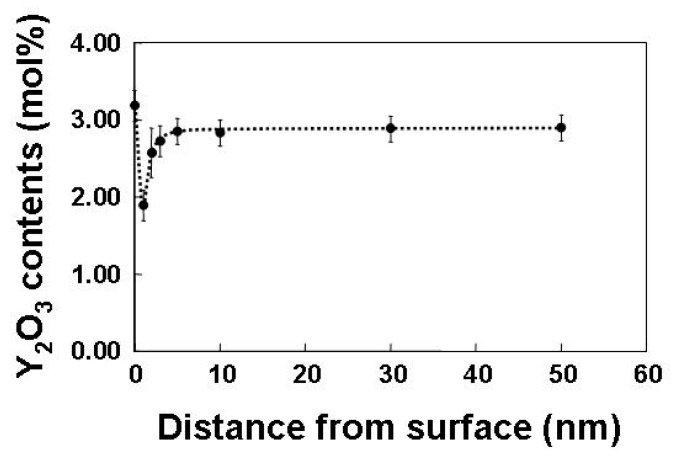
A composition of yttria estimated by quantitative analysis of EDS spectra in yttria doped zirconia particle as a function of the distance from the surface. Reprinted from Ref. [51] with permission from John Wiley and Sons.

Figure 4 shows the Y_2_O_3_ distribution in a single particle, which is quantitatively analyzed using a point analysis. This figure presents the surface segregation of Y_2_O_3_. The Y_2_O_3_ content decreases steeply to a few nanometers from the surface, and the contents are recovered up to a nominal composition of approximately 3 mol% within less than 10 nm from the surface, and they remain inside the particles. This chemical inhomogeneity remains as grain boundary segregation after sintering [52], and it plays an important role in the phase transformation in the ZrO_2_-Y_2_O_3_ system. In these ZrO_2_ particles, a monoclinic region is observed near the surface, in which both phases exhibit a specific orientation relationship. The occurrence of the monoclinic phase is attributed to the stress-induced transformation during sample preparation [53,54,55,56,57,58]. In this study, a quantitative analysis is performed on the composition distribution of the nanoparticle surface segregation.

## 3. Experimental Characterization of the Local Magnetic Moment by TEM-EELS and Its Grain Boundary Character Dependence

In Section 2, the basis of EDS and chemical analysis by EDS is described with a case study of solid–vapor interface segregation in ZrO_2_ nanoparticles within a few nm from the surface as an example of the chemical analysis, such as the chemical composition and distribution. On the other hand, in EELS, the energy distribution of the electrons, which results in inelastic interactions during the penetration of the specimen, is analyzed for the electronic structure at a high spatial resolution and used as the characterization of the chemical bonding rather than the chemical composition [59]. The detected peaks are categorized into zero loss, plasmon loss, and core loss peaks, which are caused by an elastic interaction (no energy loss), inelastic interaction with valence electrons, and electrons in the inner shell. The characterization of the electronic structure by combining EELS analysis and ab initio calculations has recently been reviewed [60,61]. In addition, EELS has the advantage of analyzing light elements such as carbon, nitrogen, and oxygen because of the high energy resolution, which is less than 1 eV and much higher than that of EDS (approximately 140 eV). A concept similar to EDS was used to perform a quantitative analysis utilizing EELS, and the relationship between the chemical amount, represented as the number of elements, and the peak intensity was expressed by

(2) $$N_{A}/N_{B}=\left\{\left(s_{A}/s_{B}\right)-1\right\}\times I_{A}/I_{B},$$

where *N*_x_, *s*_x_, and *I*_x_ represent the number of elements x, partial ionization cross section of x, and peak intensity of x above the background, respectively. This equation can be used to perform a quantitative analysis using EELS. However, EDS is frequently used for quantitative chemical analyses because of the high background of the energy loss spectrum and multiple scattering depending on the specimen thickness. A detailed description of the EELS is provided in the references [59,62]. Although quantitative analysis by electron energy loss spectrum is performed for estimating the specimen thickness using the zero-loss peak, etc., there is a report on the relationship between the d-state occupancy in transition metals, and the intensity ratio of the two L edges referred to the white line [63,64]. Pease et al. proposed a relationship between the white line ratio and magnetic moment [65]. Based on their report, we demonstrated the quantitative measurement of the local magnetic moment at the grain boundary in 3D transition metals [66,67,68,69].

Goodenough pointed out the possibility of a nucleation center for the domains of reverse magnetization in ferromagnetic materials [70] to determine the relationship between magnetism and grain boundaries; many researchers have reported these relationships, such as the interaction between the magnetic domain wall and grain boundary [71,72,73,74,75,76] and theoretically [77,78,79,80,81,82,83,84,85]. Recent ab initio calculations demonstrated the grain boundary magnetism that the local magnetic moment increases at the grain boundary; this increase significantly depends on the grain boundary geometry (e.g., misorientation angle) and chemistry (grain boundary segregation) [82,84,85]. However, only a few experimental studies on grain boundary magnetism have been performed, with the exception of neutron diffraction [86]. In this study, the EELS technique proposed by Pease [65] was applied to measure the local magnetic moment at the grain boundary.

**Figure 5:**
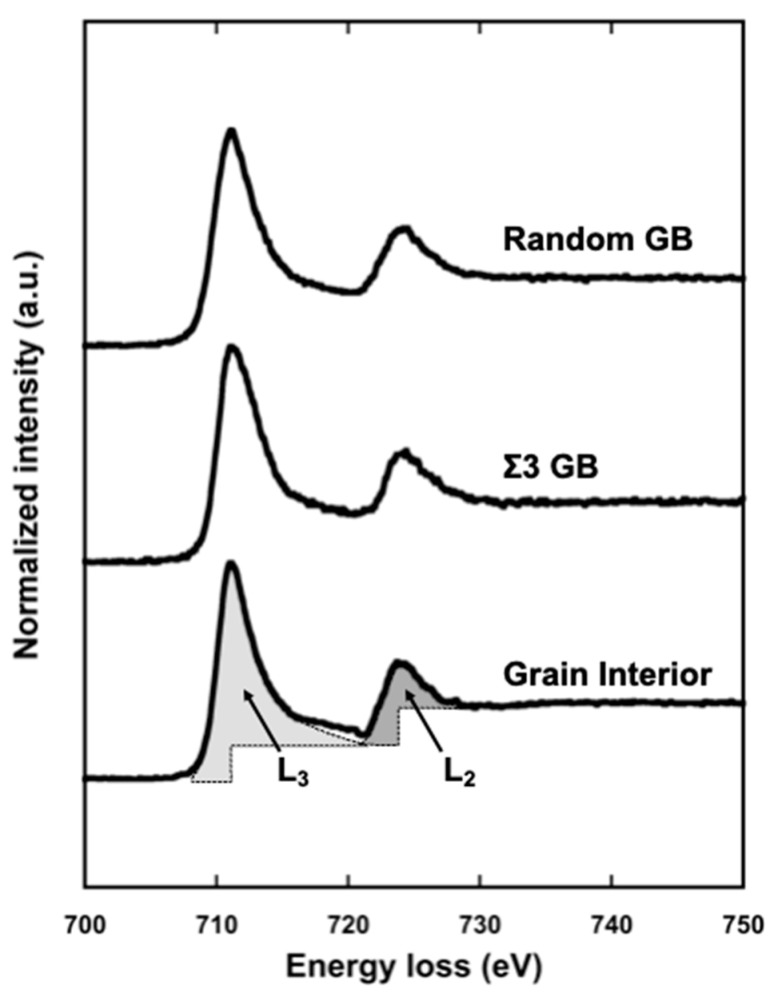
Typical L edge in EELS spectra obtained from grain interior (bottom), Σ3 grain boundary (middle), and random grain boundary (top) in iron, respectively. The L_2_ and L_3_ absorption edges are clearly seen at 710 eV and 725 eV, respectively. Each hatched region shown in the L_2_ and L_3_ edge represents schematically the line intensity in L edge obtained from grain interior, which is used in calculating the white-line ratio, R. Reprinted from Ref. [67] with permission from Elsevier.

**Figure 6:**
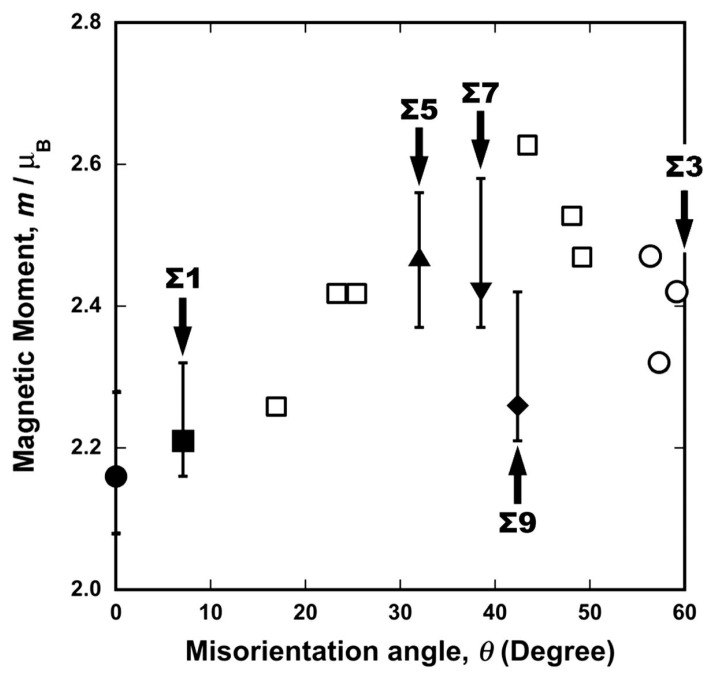
Experimentally obtained local magnetic moments in pure iron as function of misorientation angle. Moments were measured by the TEM–EELS technique. Solid circle, square, triangles, inverse triangle, and rhombus correspond to the grain interior, Σ1, Σ5, Σ7, Σ9 coincidence boundaries, respectively, and open circles and squares to the Σ3 and random boundary. Reprinted from Ref. [67] with permission from Elsevier.

Figure 5 shows an example of the electron energy loss spectra of the Fe–L edge obtained from the grain interior (bottom), Σ3 grain boundary (middle), and random grain boundary (top) in pure Fe (99.99 mass% purity). The geometry of the grain boundaries analyzed by EELS was characterized by orientation image microscopy (OIM) with electron backscatter diffraction (EBSD). Further, in the case of TEM, information on both the grain boundary and grain interior was included even if the probe had a diameter of less than 1 nm. The technique proposed by Gu et al. [87,88] was used to extract components only from the grain boundary. The measured L_3_/L_2_ ratio, termed the white-line ratio, is summarized as a function of the misorientation angle in Figure 6. The magnetic moment at the grain boundary was enhanced at a high misorientation angle compared to that in the grain interior. Furthermore, the cusp of the magnetic moment can be observed at the S9 boundary. This is the first report on the grain boundary character dependence of local magnetic moments. The grain boundary is the preferred site for segregation, and therefore, the effect of grain boundary segregation on magnetism has been widely examined [84,85,89,90,91,92,93,94,95,96,97]. The effect of grain boundary segregation on the local magnetic moment was investigated.

**Figure 7:**
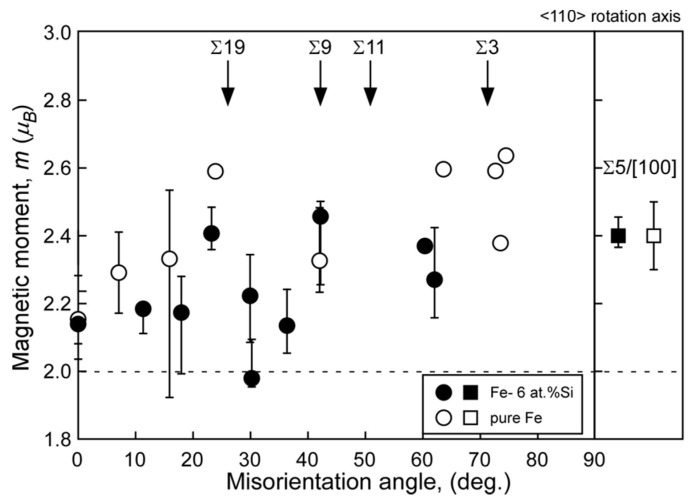
Local magnetic moments at grain boundaries in the Fe-6 at% Si alloy (solid) and pure Fe (open) as a function of misorientation angle around the <110> rotation axis. For comparison, the local magnetic moment at Σ5 grain boundary with <100> rotation axis in the Fe-6 at% Si alloy are also shown in the figure. The error bar is also shown, and data points that indicate no error bar are data points taken from single EELS measurement. The broken line in this figure indicates the saturated magnetic moment evaluated by a nuclear magnetic resonance (NMR) for the Fe-6 at% Si alloy. Reprinted from Ref. [69] with permission from the Japan Institute of Metals and Materials.

**Figure 8:**
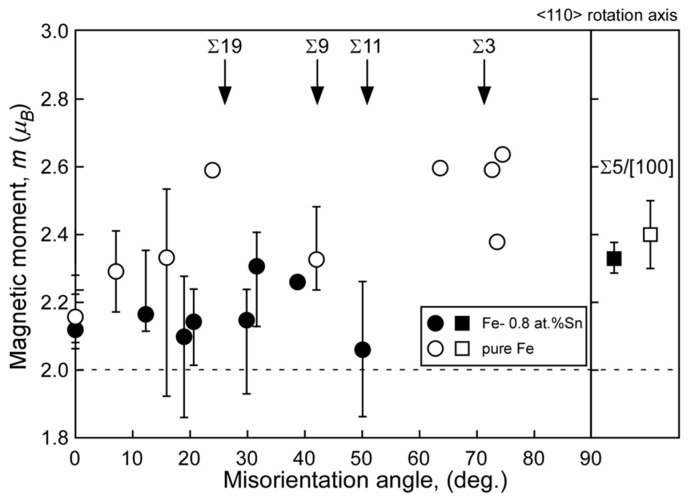
Local magnetic moments at grain boundaries in the Fe-0.8 at% Sn (solid) alloy and pure Fe (open) as a function of misorientation angle around the <110> rotation axis. For comparison, the local magnetic moment at Σ5 grain boundary around <100> rotation axis in the Fe-0.8 at% Sn alloy is also shown in the figure. The error bar is also shown, and data points indicating no error bar are data points taken from single EELS measurement. The broken line in this figure indicates the saturated magnetic moment evaluated by a vibrating sample magnetometer (VSM) for the Fe-0.8 at% Sn alloy. Reprinted from Ref. [69] with permission from the Japan Institute of Metals and Materials.

**Figure 9:**
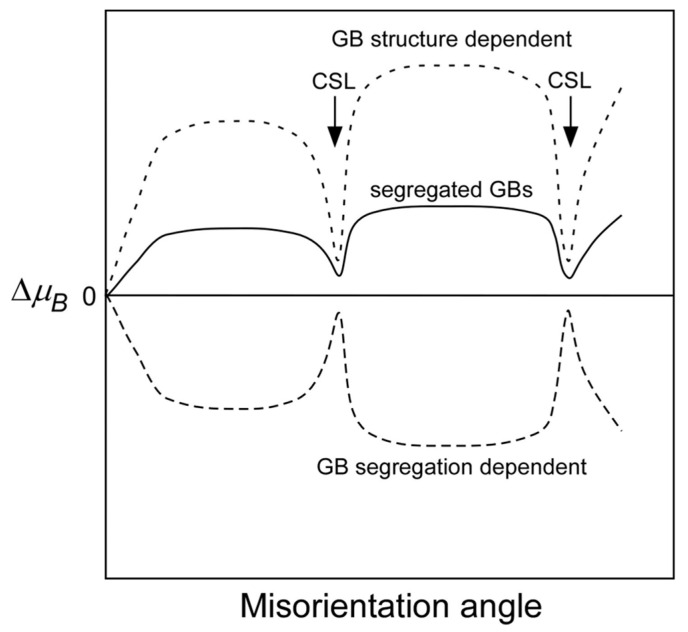
Schematic explanation of the misorientation dependence of the local magnetic moments at solute (impurity)-segregated grain boundaries, which will be determined by competing between the favoring effect due to the GB structure and disfavoring effect due to grain boundary segregation on the local magnetic moments. Reprinted from Ref. [69] with permission from the Japan Institute of Metals and Materials.

Figure 7 and Figure 8 show the misorientation-angle dependence of the local magnetic moment obtained in Fe-6 at% Si and Fe-0.8 at% Sn alloys [69]. Both figures include the magnetic moment at the grain boundary obtained for pure Fe as a reference [67]. The local grain boundary moment at the random boundary in the Fe–Si and Fe–Sn alloys was reduced compared to that in pure Fe. However, there is no remarkable change at the Σ5 boundary. These results suggest that the local magnetic moment is determined by combining the physical effect of the geometry and the chemical effect of segregation, as illustrated in Figure 9.

To characterize the magnetic properties by the L edge, X-ray magnetic circular dichroism (XMCD) has been well known [98,99,100,101]. On the other hand, Schattschneider et al. proposed electron magnetic circular dichroism (EMCD) to characterize them in terms of (S)TEM and EELS [102,103,104], and several researchers conducted it [105,106,107]. Muto et al. evaluated the ratio of the orbital (m_l_) and spin (m_s_) magnetic moments (m_l_/m_s_) [106]. The details of magnetism are clarified at a high spatial resolution by combining these techniques. Further, grain boundary segregation is one of the main factors of grain boundary embrittlement [108,109]; recent ab initio calculations represented the effect of grain boundary segregation and resulting in the local magnetism at the grain boundary on grain boundary embrittlement in Fe–Mn alloys [110]. They also discussed embrittlement from the viewpoint of changes in magnetism at the grain boundary. The relationship between grain boundary segregation and grain boundary embrittlement in steel was reported approximately half a century ago [111,112], and state-of-the-art experiments and theoretical calculations are expected to answer this question.

## 4. Dynamics and Quantitative Evaluation Associated with the Grain Boundary by In Situ Experiments

The above two sections represented the quantitative analyses by EDS and EELS combined with TEM. EDS and EELS analyses are usually conducted statically because of the time-consuming techniques for signal detection, whereas understanding the dynamics of the materials in external fields is also significantly important to comprehend how response in the materials. The in situ experiment in TEM and SEM is a powerful tool for clarifying the behavior of materials for the various fields, and one can see many reports on the response of the materials under various fields, such as temperature, stress, and atmosphere [113,114,115]. On the other hand, since the publication of the reports by Hall [116] and Petch [117], the Hall–Petch relationship has been well-known for explaining the relationship between mechanical properties and grain size in polycrystalline materials. The grain size corresponds to the grain boundary density; in other words, the mechanical properties are governed by the grain boundaries. The pile-up model is widely accepted for understanding the Hall–Petch relationship [118], and the grain boundary–dislocation interaction has been investigated from both experimental and computational viewpoints [119,120,121,122,123,124,125,126,127,128,129,130,131,132,133,134,135,136,137,138,139,140,141,142,143]. Recently, nanoindentation techniques have been employed to understand the mechanical response in the vicinity of an individual grain boundary [128,129,130,131,132,133,134,135,136,137,138,139,140,141,142,143]. Moreover, in situ TEM (and SEM) straining experiments are powerful tools for understanding the microstructure evolution of small-scale specimens under the stress field. These have received considerable attention for deformation in the grain interior [144,145,146,147,148,149,150,151,152,153,154,155,156,157,158,159], deformation [160,161,162,163,164,165,166,167,168,169,170,171,172,173,174], and fracture [175,176,177,178] around grain boundaries. Among them, some specimen holders can explore the simultaneous mechanical behavior during the microstructure evolution [146,147,150,151,152,154,156,157,159,160,161,162,163,164,165,166,167,168,169,170,171,172,173,174].

**Figure 10:**
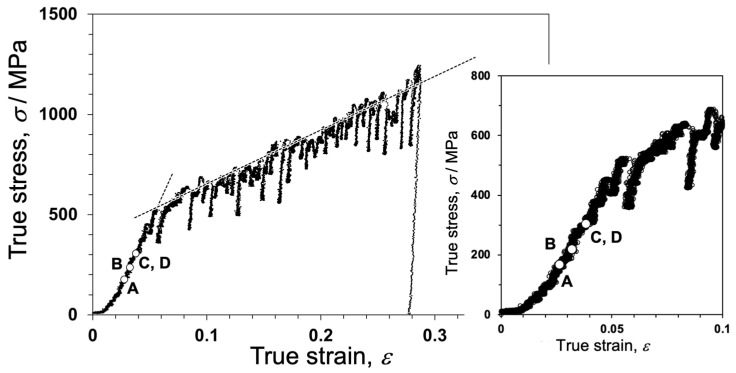
True stress–true strain (S–S) curve of the bicrystal pillar, including the Σ3 grain boundary. The dashed black lines evaluating the pseudo yield stress in the bicrystal are also drawn in the S–S curve. The initial stage of the S–S curve is also shown. To demonstrate the accuracy of the in situ TEM experiment, S–S curves of the single crystal pillars obtained from each grain composing the bicrystal are shown with this S–S curve in Figure S1 in the supplementary material in Ref. [173]. Reprinted from Ref. [173] with permission from Elsevier.

**Figure 11:**
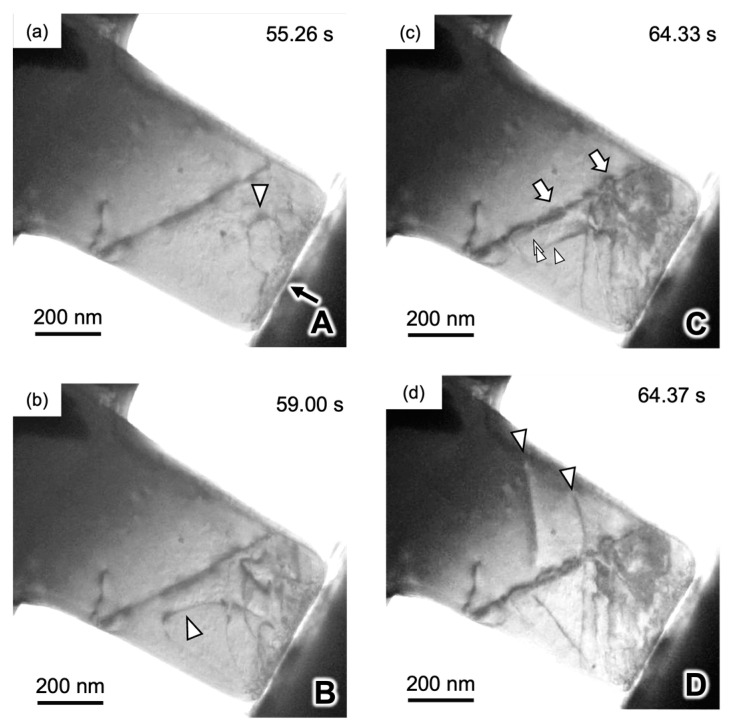
(**a**–**d**) Snapshots captured the movie during compression for the Σ3 bicrystal pillar. A–D inserted into the lower right on each image correspond to points (A–D) indicated in the S–S curve in Figure 2, respectively. Dislocations indicated by triangles are activated from the contact between indent and sample in the grain A. Details can be represented in Ref. [173]. Reprinted from Ref. [173] with permission from Elsevier.

Figure 10 shows the true stress–true strain (S-S) curve of a 4N Al bicrystal pillar, including a single Σ3 grain boundary, obtained by in situ indentation experiments. The S–S curve exhibits an apparent yield phenomenon around the crossing point of the two dashed lines drawn in the S–S curve, and after yielding, a small stress drop is frequently detected. This phenomenon is known as intermittent plasticity [156,179]. Figure 11 shows snapshots captured in the movie during in situ straining for the Σ3 bicrystal pillar. This movie is available as a supplementary movie in Ref. [173]. Figure 11a,d correspond to A to D in Figure 10, respectively. The dislocations were activated and transferred across the grain boundaries before the macroscopic yield phenomenon. The critical resolved shear stress (CRSS) that the dislocation is transferred across the grain boundary was measured from the S–S curve in Figure 10 within 0.06–0.09 GPa. This CRSS matches the stress range estimated from the Hall–Petch coefficient obtained from the macroscopic experiments [180,181].

**Figure 12:**
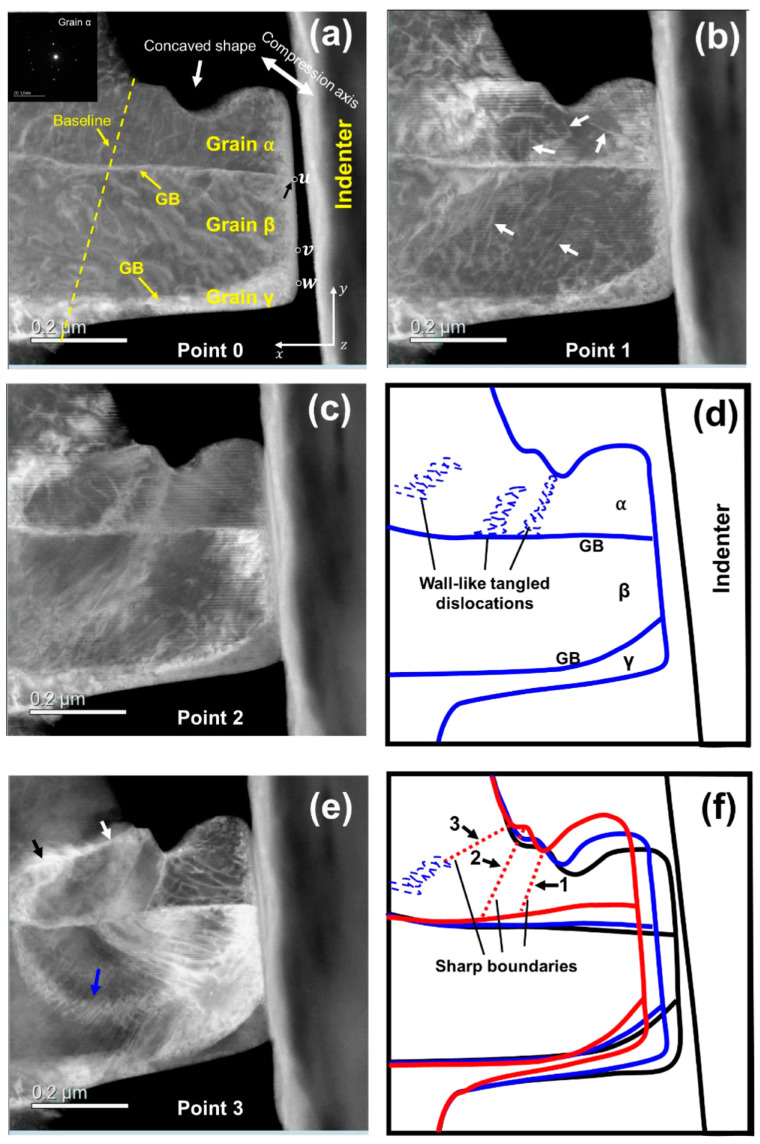
Dynamic observation of GB formation in bcc iron during TEM in situ compression testing. Low-angle annular dark-field (LAADF)- STEM micrographs of the micropillar (**a**–**c**,**e**), corresponding to points 0–3 in Figure 1 of Ref. [159], respectively. (**d**) Illustration of the wall-like tangled dislocations in (**c**). (**f**) Illustration of the formed GBs and the changes in the geometry of micropillar. The black, blue, and red outer contours represent the geometries of the micropillar in (**a**,**c**,**e**), respectively. The inset in (**a**) is a selected area electron diffraction pattern of grain α before deformation. Details can be represented in Ref. [159]. Reprinted from Ref. [159] with permission from Elsevier.

**Figure 13:**
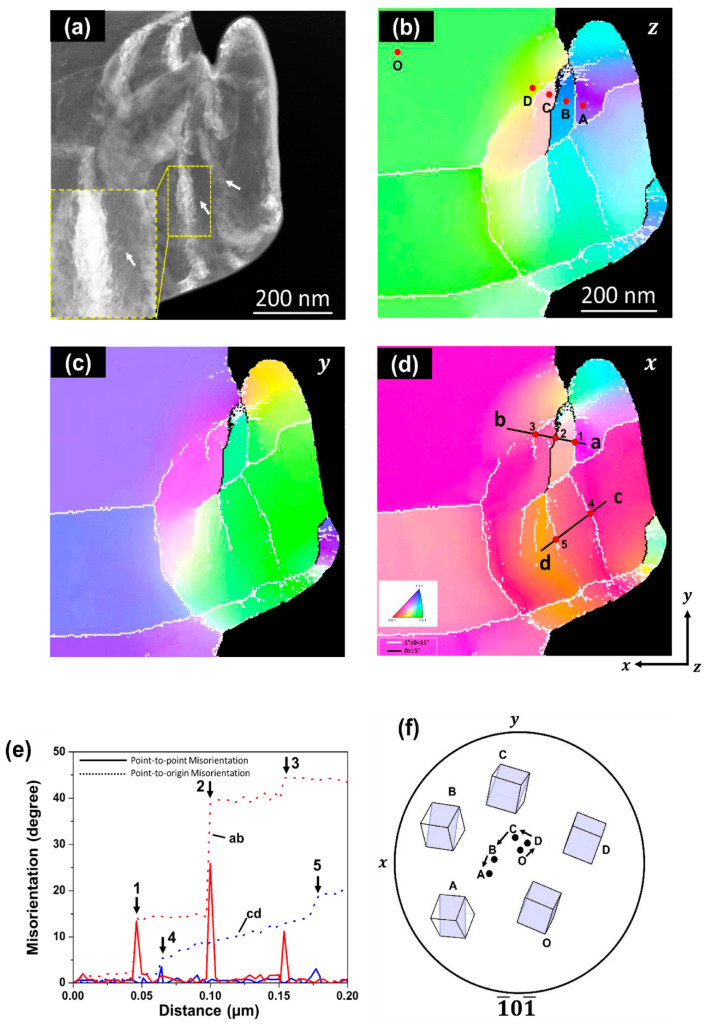
GB confirmation by precession electron diffraction. (**a**) LAADF-STEM of micropillar after TEM in situ compression test. Orientation maps obtained by scanning precession electron diffraction measurements from (**b**) z direction, (**c**) y direction, and (**d**) x direction. (**e**) Misorientation line profile of line segments ab and cd in (**d**). (**f**) The crystallographic orientations of the positions of O and A–D are plotted in the $\left(\bar{1}0\bar{1}\right)$ pole figure. The orientation at position O at the grain center was used as the original orientation of grain α. In these orientation maps, the grain boundaries with misorientation angles (θ) of 2° ≤ θ ≤ 15° and 15° ≤ θ are defined as low- and high-angle boundaries, which are indicated as white and black lines, respectively. Details can be represented in Ref. [159]. Reprinted from Ref. [159] with permission from Elsevier.

Further, it also succeeded in capturing grain boundary formation attributed to the dislocation accumulation via in situ straining experiments [159]. Figure 12 shows the dynamics of grain boundary formation in the IF steel during in situ straining. Stress inhomogeneity occurred because the specimen included a concave surface, which resulted in the activation of various slip systems. Tangled dislocations subsequently formed sharp grain boundaries, as shown in micrograph (e) and schematic (f). The line contrast was experimentally recognized as a grain boundary by automated crystal orientation mapping (ACOM) [182], as shown in Figure 13. The orientation of the deformed specimen indicated in the Low-angle annular dark-field (LAADF)-STEM image (a) is analyzed as (b–d), which are imaged in the z (b), y (c), and x directions (d). The coordinates are indicated at the bottom left in (d). Further, the grain boundaries determined from the crystal orientation are represented by black and white lines. The white line corresponds to misorientation angles from 2° to 15°, and the misorientation angle of the black line is greater than 15°. The white and black lines represent the small- and high-angle grain boundaries, respectively. In this case, the misorientation angle of each grain boundary, indicated as the line between a to b and c to d in (d), is evaluated in (e) and quantitatively understood as the existence of sharp peaks or steps in (e). The grain boundary formation can be explained by the dislocation reaction. The grain boundary formation through deformation is recognized as grain refinement in ultrafine-grained metals via severe plastic deformation [183,184]. Also, grain refinement is achieved via grain subdivision during deformation [185,186,187,188,189]. The microstructural evolution obtained in this study is well explained by grain boundary formation. A similar phenomenon was reported in [190]; however, it was observed for a nanowire. This is the first report of a specimen that is close to the bulk scenario. The deformation microstructures in various nanocrystalline materials were also quantitatively analyzed by utilizing a combination of ACOM and in situ straining experiments [191,192,193].

## 5. Prospects of the Quantitative Characterization of Interface and Grain Boundary by (S)TEM

In the previous sections, it was shown that the quantitative analysis performed by EDS and EELS combined with (S)TEM and related applications to the interface and grain boundaries with the obtained examples. However, new analytical techniques are still being developed for understanding unknown issues of the interfacial phenomena.

The first is an improvement in the accuracy of the quantities. As aforementioned in the first section, STEM is commonly applied to the characterization of the microstructure and the spectroscopic analysis. It is well known that STEM images are generated by the detection of electrons transmitted during the scanning of the specimen, and there are many types of images depending on the interaction between the electron and specimen [194]. High-angle annular dark-field (HAADF) imaging is used to detect scattered electrons at a high angle through the specimen, and the intensity is closely related to the atomic number (Z). Furthermore, the bright contrast in the HAADF image corresponds to the atomic column, and the interpretation of the images is more straightforward than that of the HRTEM images. Therefore, STEM has become a standard technique for characterizing atomic structures. In STEM images, the key to improving spatial resolution is the formation of a probe using an aberration correction technique; currently, a spatial resolution of up to 40.5 pm has been realized [195]. Furthermore, the spatial resolution of spectroscopy is also improved with the fine probe, resulting in the chemical mapping at an atomic scale by EDS and EELS with STEM have been successfully obtained so far [43,44,45,46,47,48,49,50,196]. Moreover, several works on estimating the chemical composition from STEM images have also been reported recently [197,198,199,200,201]. Van Aert et al. [197] and Molina et al. [198] proposed estimating the composition from the intensity of each column in the HAADF-STEM images. Subsequently, the composition of the Al matrix, the vacancies in the Al alloys [199], and the absolute value from the column composition were estimated. These techniques were improved by combining them with image simulations [200]. Recently, the light elements were also quantitatively analyzed [201]. For the further improvement of the chemical analysis, understanding the quantitativeness caused by an interaction of the electron and specimen is important.

On the other hand, chemical and physical properties, such as the grain boundary segregation and the grain boundary energy, are affected by the inhomogeneity of the atomic structure. The inhomogeneity is closely related to the excess volume at the grain boundary. Branova [202] attempted to analyze the excess volume at the grain boundary using HAADF-STEM images. The excess volume is also detected as the local elastic strain. The techniques for the quantitative evaluation of the displacement of atoms and related elastic strain developed by Hÿtch [203,204] and Galindo [205] provided remarkable progress in understanding the strain distribution in the microstructure, especially on the atomic scale. Thus far, there have been many reports on the characterization of elastic strain not only at the grain boundaries as twins [206] and dislocations at the grain boundaries [207,208,209] but also at the interphase boundary in the device [210], heterointerface at an epitaxially grown superlattice [211], and precipitate/matrix interfaces [212,213,214,215,216]. In addition to the strain (stress) field evaluated by the precise measurement of the displacement of atoms, the differential phase-contrast (DPC) imaging by a segmented detector [217] spread a new era in the visualization of electric [218,219] and magnetic fields [220] on an atomic scale. This technique is currently used to characterize heterointerfaces in a functional device [221,222,223].

A novel quantitative analysis of the physical properties using the EELS is also proposed [224,225,226,227]. The core loss edges in EELS are frequently used to analyze the physical and chemical properties related to electrons. In contrast, Oleshko et al. proposed the use of plasmon loss peaks, termed valence EELS (VEELS), to characterize the physical properties of the interphase boundary [224]. Subsequently, Nandy et al. applied VEELS to grain boundaries and discussed the correlation between VEELS and the electron density and volume expansion of the grain boundary [225,226,227]. From a theoretical perspective, Kohyama et al. evaluated the local energy and local stress at the surface and grain boundaries using ab initio calculations [228]. The local energy can be quantitatively evaluated by combining these techniques. Further, a low-loss region was used to characterize the surface plasmons [229] and quantitatively evaluate the bandgap near the Si/SiO_2_ heterointerface [230]. Further, the remarkable improvement in the energy resolution in EELS using a monochromated electron probe was addressed by Krivanek [231], and a recent energy resolution of less than 10 meV was achieved. This improvement enabled us to evaluate the vibrations, especially the localized vibrational response demonstrated at the heterointerface [232] and grain boundary [233].

In contrast to the analytical viewpoint, the grain boundary engineering proposed by Watanabe in the 1980s from the standpoint of material science [25,26] made a trend to tailor the grain boundary microstructure, which is focused on the geometry of grain boundaries such as the misorientation angle and S-value, termed a “grain boundary character”. In addition to Watanabe’s concept, Raabe proposed “grain boundary segregation engineering” in his review [234] and attempted to control the chemistry of the grain boundary. This concept has spread to the interphase boundary [235] and has been demonstrated [236,237,238]. Atom probe tomography is an important tool for understanding the chemical distribution at specific interfaces and boundaries and the relationship between structure and chemistry [234,235,236,237,238,239,240,241]. However, quantitative analysis using electron microscopy is indispensable because simultaneous observations and analyses can be performed. Furthermore, an in situ study of the mechanical behavior on an atomic scale was performed using a precise MEMS system [242]. The continuous developments of the aforementioned analytical techniques for hardware and software are expected to help elucidate the interfacial phenomena that remain unexplored, which is a direct measurement of the interfacial energy and their distribution in the metastable state/matrix interphase boundary, which only (S)TEM can explore their microstructure, etc.

## 6. Concluding Remarks

In this article, the quantitative analysis by EDS and EELS equipped with the TEM is described, along with its application to the phenomena accompanied by the interface and the grain boundary. As seen in the references, it is challenging to summarize because of the significant work on the characterization of the interface and the grain boundary, but the points of this article are concluded as follows,

1. EDS analysis, which is frequently used in chemical analysis, such as the identification of the elements and the chemical compositions, was explained. Based on this, the solid–vapor (surface) segregation of doped Y_2_O_3_ within a few nm from the surface in ZrO_2_ nanoparticles was explored.
2. The TEM-EELS technique, which is often used to understand the electronic structure, is conducted to characterize the local magnetic moments closely related to 3D electrons. Also, the grain boundary character dependence and the effect of the grain boundary segregation of the magnetic moments are quantitatively obtained.
3. The characterization of the dynamics of materials by an in situ experiment is also presented. The in situ straining experiments succeeded in the direct stress measurement at the dislocation transfer to the grain boundary and the capture of the microstructure evolution to form grain boundaries during plastic deformation.
4. In addition to the application of EDS and EELS to characterize the interface and grain boundary that the author reported so far, the prospects of the characterization of the interface and grain boundary are stated for further improvement of the quantitative analysis.

## Data Availability

The authors of this paper used the collections of journals and electronic databases available in Scopus.
